# Machine Learning Methods for Control of Fibre Lasers with Double Gain Nonlinear Loop Mirror

**DOI:** 10.1038/s41598-019-39759-1

**Published:** 2019-02-27

**Authors:** Alexey Kokhanovskiy, Aleksey Ivanenko, Sergey Kobtsev, Sergey Smirnov, Sergey Turitsyn

**Affiliations:** 1 Novosibirsk State University, Pirogova 2, Novosibirsk, 630090 Russia; 2 Aston Institute of Photonic Technologies, Aston University, B4 7ET Birmingham, UK

## Abstract

Many types of modern lasers feature nonlinear properties, which makes controlling their operation a challenging engineering problem. In particular, fibre lasers present both high-performance devices that are already used for diverse industrial applications, but also interesting and not yet fully understood nonlinear systems. Fibre laser systems operating at high power often have multiple equilibrium states, and this produces complications with the reproducibility and management of such devices. Self-tuning and feedback-enabled machine learning approaches might define a new era in laser science and technology. The present study is the first to demonstrate experimentally the application of machine learning algorithms for control of the pulsed regimes in an all-normal dispersion, figure-eight fibre laser with two independent amplifying fibre loops. The ability to control the laser operation state by electronically varying two drive currents makes this scheme particularly attractive for implementing machine learning approaches. The self-tuning adjustment of two independent gain levels in the laser cavity enables generation-on-demand pulses with different duration, energy, spectral characteristics and time coherence. We introduce and evaluate the application of several objective functions related to selection of the pulse duration, energy and degree of temporal coherence of the radiation. Our results open up the possibility for new designs of pulsed fibre lasers with robust electronics-managed control.

## Introduction

Increasing complexity is an emerging trend in modern engineering systems. Many factors contribute to the complexity and uncertainty of system behaviour, including noise, nonlinear dynamics, multi-parametric operational space and changing environments. Complexity imposes extra requirements on control system design in terms of maintaining accuracy; fast adaptation to changing environments; and variations in operational regimes. Most classical control approaches have been designed for linear systems and are often insufficient in complex nonlinear systems. In this context, machine learning (ML)-based approaches offer a nonlinearity-friendly, efficient and flexible alternative to the classical control techniques. ML-based control methods have been studied extensively and have proved indispensable in systems where knowledge of the underlying mathematical models is limited or absent. In the field of lasers, the recent pioneering works of the N. Kutz group^1–3^, which combined ML techniques with adaptive control, have demonstrated that robust, self-tuning operation can be achieved in nonlinear polarization evolution (NPE)-based mode-locked lasers. This a transformative concept because while NPE-based mode-locked lasers demonstrate excellent performance^4,5^, the reproducibility and control of the operation of such lasers is a major technical challenge due to multiple operational states of the corresponding nonlinear systems^6,7^. Several groups^1–3,8–14^ have recently demonstrated that the performance of NPE-based mode-locked fibre lasers can be efficiently optimised using ML methods. It has also been shown that the NPE-based method of a controlling the pulse parameters can be implemented in combination with other mode-locking techniques, for example, using a nonlinear amplified loop mirror. Flexible self-tuning lasers have the potential to revolutionise both the laser industry and the research applications of lasers.

The key factor that contributes to the complexity of fibre laser systems is the underlying nonlinear dynamics of light (see, e.g. ^15–18^ and references therein). The generation and subsequent shaping of the laser radiation is determined by a complex interplay between physical effects, such as nonlinear gain and loss, fibre dispersion and Kerr nonlinearity, and filtering. The comprehensive understanding of the fundamental nonlinear science underlying the operation of fibre lasers is a challenging physical problem. Modelling can provide a theoretical framework for understanding some of the features of mode-locked fibre lasers, but achieving a one-to-one mapping between the numerical modelling and experiments is a problem due to a limited knowledge of the various system parameters and model limitations. It is important to recognise that although the fibre nonlinearity creates a difficulty with understanding the laser system operation; this same nonlinearity provides the conditions for mode locking and makes it possible in the first place. Machine learning techniques can offer a path to make controllable positive use of these nonlinear effects.

The present work goes beyond the NPE-based lasers and introduces ML-based control to a new class of mode-locked fibre lasers with a double-gain, nonlinear amplifying loop mirror (modification of the figure-8 lasers^19–21^). This system offers the possibility of combining simple electronic control of the nonlinearity and pulse properties^22^ with ML algorithms and objective functions. We should stress that the novelty of using a ML approach in this scheme is not limited to scaling up pulse parameters and optimisation of the laser performance. Application of ML methods provides the opportunity to generate on-demand, qualitatively new, lasing regimes by choosing the appropriate objective function. Employing ML techniques can also assist in the development of new advanced laser systems in several ways. First, at the design and testing stage, ML can provide a new level of comprehension of the lasing regimes that are available for a given system configuration and how these regimes will be affected by numerous possible modifications that can dramatically expand the performance of a laser. This new feature cannot be practically implemented without ML. Second, an important new element is that, with simplified processing of the generated signal, ML-based lasers can potentially be self-tuned and extremely robust against various environmental perturbations and hazards. Achieving this feature will require further studies; however, we consider the present paper to be an important step in this direction.

Here, for the first time, we apply ML methods to control the radiation of a fibre laser with two electronic-managed distributed gain parameters; this makes it possible to effectively control the onset of the nonlinear dynamics of optical radiation in the laser cavity and to obtain pulsed regimes with different duration, energy, optical spectrum width and degree of coherence. We introduce a number of objective functions that make it possible to generate on-demand pulses with the shortest duration; the maximum energy; and to vary the degree of coherence. The flexibility of the proposed laser scheme is further demonstrated for implementing algorithmic, electronically-driven control over radiation mode-locking regimes.

## Experimental Setup

**Figure 1:**
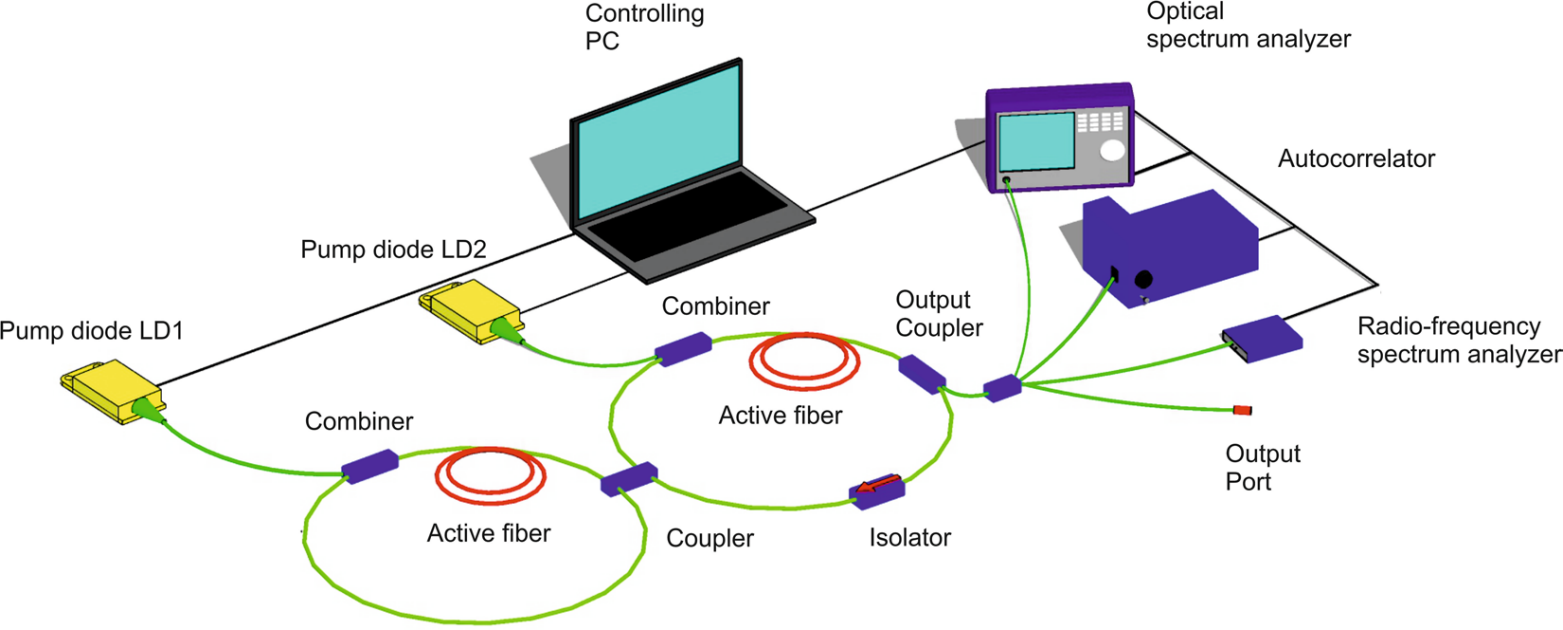
Schematic of a fiber laser with two active stretches of fiber in both loops.

We consider the scheme of the figure-eight, mode-locked, fibre laser cavity (see Fig. 1 and Methods). The cavity consists of two fibre loops, left (unidirectional) and right (bidirectional), which are connected to each other by a 40/60 coupler. Both loops of the laser resonator contain amplifying sections pumped by multimode laser diodes. The laser cavity consists of only polarization maintaining elements to prevent nonlinear polarization evolution effects. Therefore, the output radiation is linearly polarized. Independent control of the currents of the two pump diodes provides significant variability of the pulsed regimes with different output average power, radio-frequency contrast, duration of autocorrelation function and degree of coherence.

An automated system for laser control, data acquisition and processing were built (see Fig. 1 and Methods) for comprehensive measurements of the parameters of the pulsed generation regimes. For diagnostics, the power was mainly determined from the autocorrelation measurements. Some part of radiation is used for feedback loop and it should be checked that the overall system performance is not affected by this. We observed that an average laser power of 10 mW is enough to build a feedback system. As we get pulses with an average power varying from 40 mW to 300 mW such reduction of output power will not play crucial role in the performance of the laser system.

**Figure 2:**
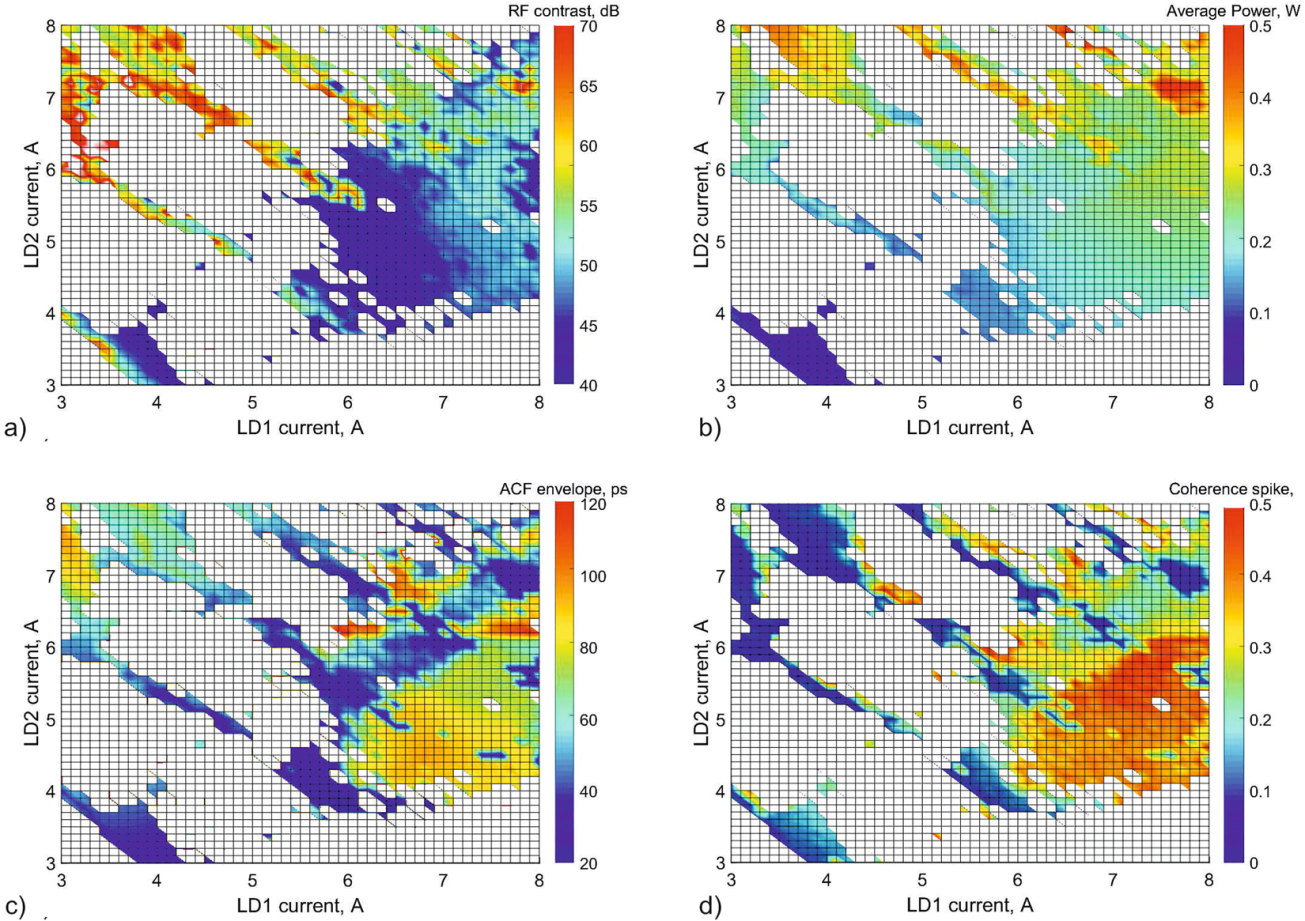
Maps of the parameters of the pulsed regimes in the plane of two currents of the pump diodes (**a**) Radio frequency contrast (dB); (**b**) Width of autocorrelation function (ps); (**c**) Average radiation power (W); (**d**) Coherence peak contrast. White colour on the maps corresponds to the absence of a mode-locked regime.

In Fig. 2, we plot two-dimensional maps of the measured RF contrast (a), average power (b), ACF envelope (c) and coherence peak contrast (d) as functions of the two pump diode currents. First, we measured the radio-frequency spectrum (RF spectrum) of the output radiation. The key parameter that indicates a mode-locked regime is a large contrast between the background level and the spike at the fundamental frequency of the laser cavity (Fig. 2a). We consider that the laser is operating in a mode-lock regime when this contrast is greater than 40 dB (more details are given below). The white colour in the maps corresponds to regimes without mode-locking (i.e. contrast < 40 dB). The second map (Fig. 2b) corresponds to an average power of the output radiation obtained by the integration of the spectral density measured by an optical spectral analyser.

In the case of double-scale pulses (or noise-like pulses), the contrast of radio-frequency spectrum decreases due to an increase of background level. This situation can be identified by analysing the pulse autocorrelation function (ACF) which provides information about the pulse duration and its degree of coherence. It is well known that the autocorrelation function of double-scale pulses exhibits a central coherence spike, the height of which indicates the degree of coherence of the mode-locking regime^7^. To measure the contrast of this coherence spike with regard to the ACF envelope, we first applied a low-pass Butterworth filter that removes the coherence spike. We used the full-width half maximum of this filtered ACF as a measure of the duration of the ACF envelope (Fig. 2c) and then calculated the ratio between maxima of unfiltered and filtered ACFs to give a measure of the contrast of the coherence spike (Fig. 2d).

The maps (Fig. 2a,b) illustrate a large variation of pulsed regimes with different duration, energy and coherence degree that could be useful in many different applications. Mapping these pulsed regimes takes about eight hours and the resulting maps change slightly under environmental changes. It is worth mentioning that the Yb double-clad amplifier that we used in our work is less stable than a single-core amplifier because of the use of multicore pump laser diodes. However, it creates the opportunity to generate a variety of picosecond pulses with an average power of several hundreds of milliwatts and with record-high energies^23^. Unfortunately, the relatively high instability of power of multi-mode pump diodes has the consequence that many of these pulsed regimes will only remain stable over a short lifetime. Therefore, simply performing a single mapping of pulsed regimes does not guarantee finding a regime, with the desired parameters, that is also stable over a long time. Here, we use a genetic algorithm to scan the various pulsed regimes in our system, following recent works where this approach was applied in NPE lasers with an anomalous dispersion^8,12^. This algorithm operates in such a way that the existence of a suitable pulsed regime is confirmed over many iterations and this results in a much more stable regime.

## Algorithm of Genetic Search

**Figure 3:**
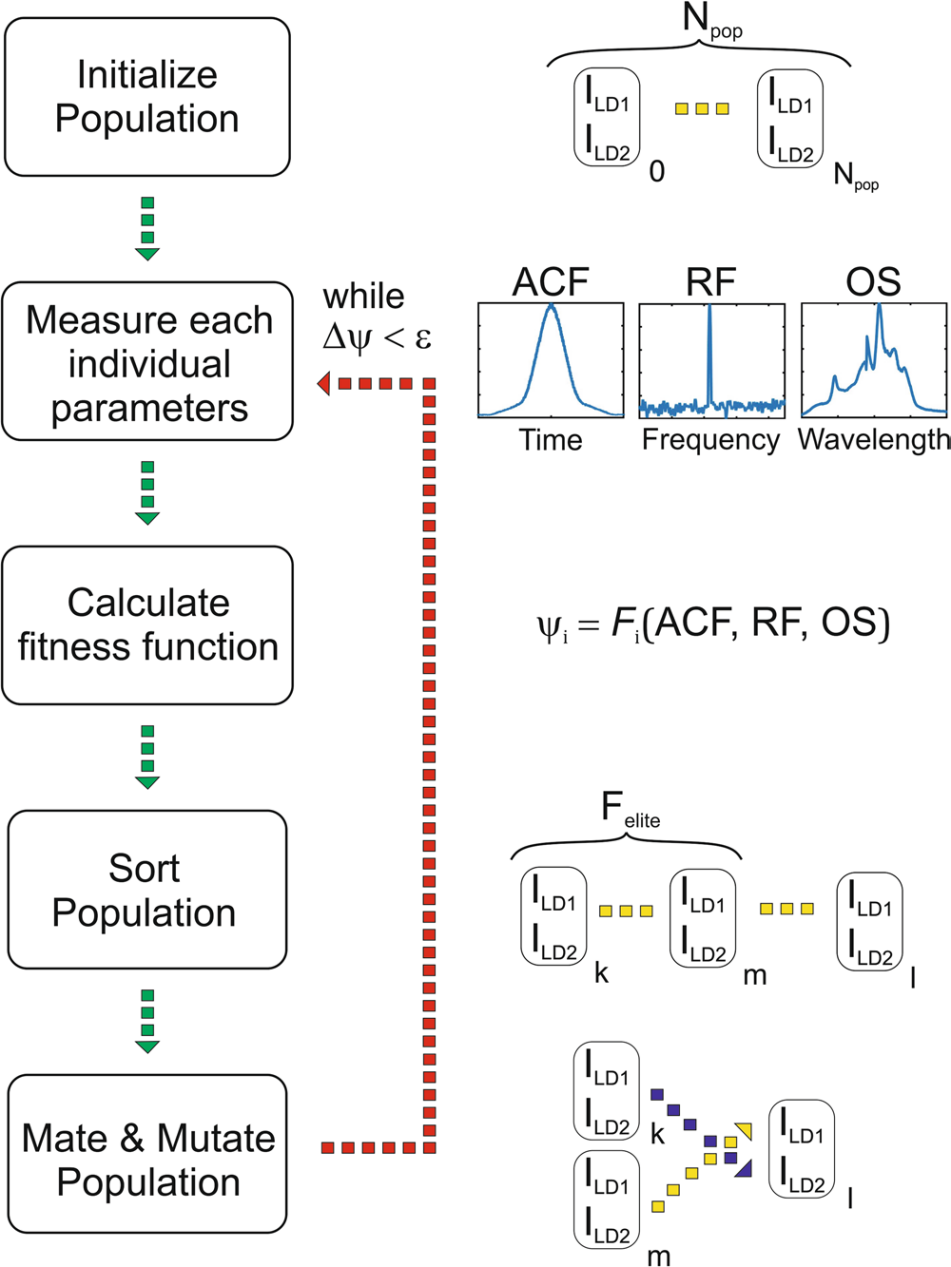
Schematic diagram of the genetic algorithm.

The schematics of the genetic algorithm used in this work is shown in Fig. 3. “An individual” of a population is a pulsed regime having two genes – which are the values of the two pumping diodes currents. Each individual has a unique set of parameters: the contrast of the RF spectrum of the fundamental mode; the average power; the duration of autocorrelation function; and the contrast of the coherence spike. By using these values, one can construct a fitness function that a genetic algorithm must optimise. The objective (fitness) function is selected in such a way that its maximum value corresponds to the pulsed regime with the targeted parameters. The genetic algorithm starts by initialising the population by assigning a random pair of currents to each individual. The pulse parameters are then measured for each individual in the population. We normalise these parameters to the maximum values in the population in order to give equal weighting to each parameter in the fitness function; thus, each value lies in the range from 0 to 1. After sorting the population by the value of the fitness function, individuals are then mated by randomly mixing their genes. The set of individuals that have the highest values of the fitness function – the elite group – are not changed. To avoid the algorithm clamping on a local maximum of the fitness function, part of the population is altered by mutation; that is, a random change of genes. The algorithm cycle can be terminated when the change in the objective function has remained less than a threshold value over several cycles. To configure the algorithm, the number of individuals in the population, the fraction of elite individuals and the fraction of mutated individuals need to be selected; these parameters are selected empirically (see Methods). It is important to note that mode-locking regimes can be affected by the hysteresis phenomena associated with the pumping power amplifying fibres. To minimise any hysteresis phenomena, the pumping currents were first reset to zero before switching to a new setting.

## Generation of the Shortest Pulse

First, we applied this genetic algorithm-based approach to generate the shortest pulses available for our fixed laser cavity. For this purpose, we used following objective function:

$${{\rm{\psi }}}_{1}=RF\,contrast+\frac{1}{ACF\,duration}$$

**Figure 4:**
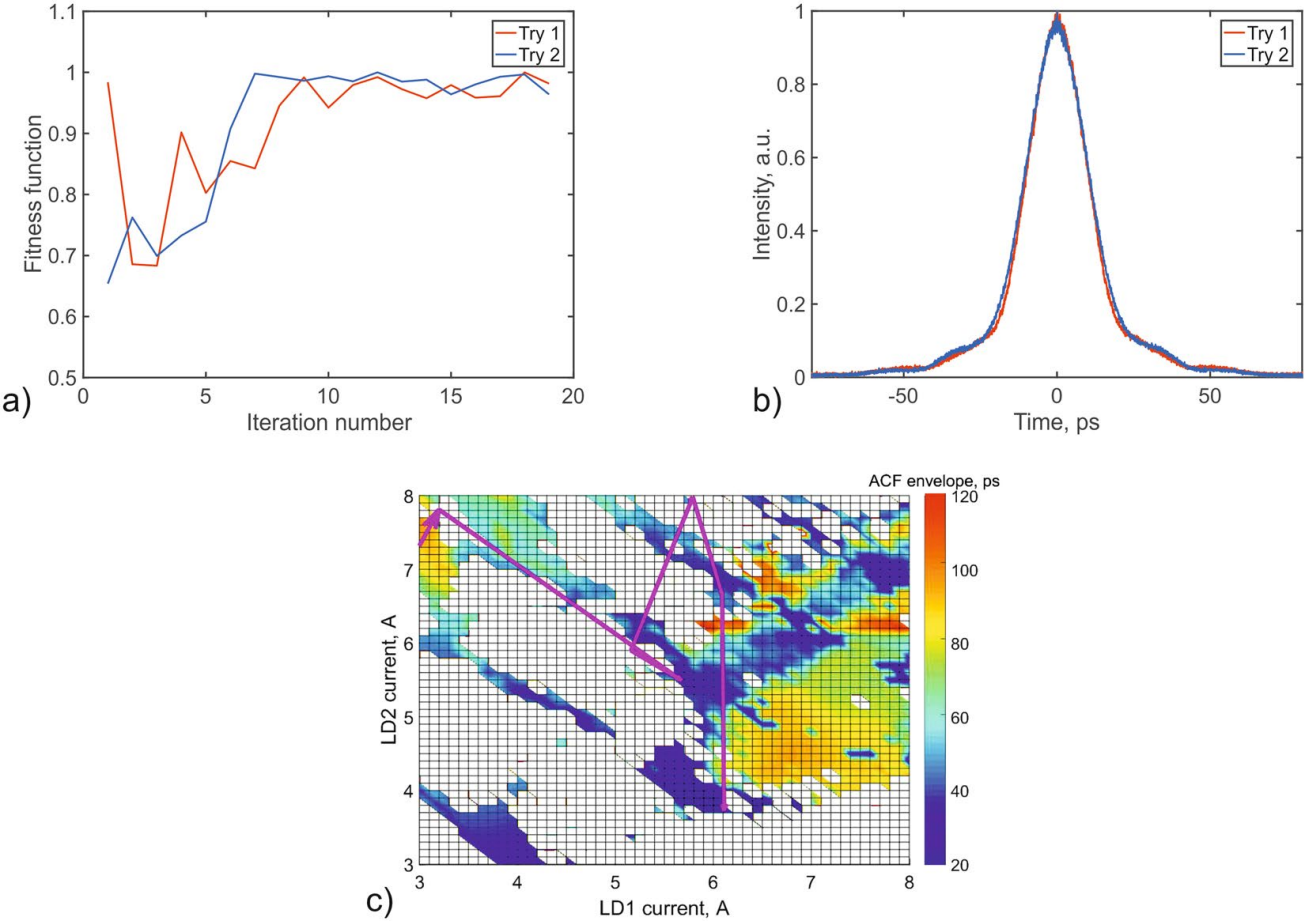
(**a**) Convergence of the objective function in the search for the pulse with the smallest duration. (**b**) ACF of the pulse with the shortest duration. (**c**) Trajectory of the best pulse in the population.

We included the RF contrast term in the function to ensure the quality of the mode-locked regime. To demonstrate the operation of this algorithm, we plot the variation of the ACF function with iteration number for two separate runs (Fig. 4a); the ACF of the final state (b); and the trajectory of the best pulse in the population (c). It takes 10–15 iterations of the algorithm to find the shortest pulses. After this point, the fluctuation of the objective function corresponds to fluctuation of the pulse parameters and does not exceed our threshold of 5%.

## Generation of the Double Scale Pulses

Double temporal scale pulses, that present either ns-scale waveforms modulated with ps-scale oscillations, or ps temporal lumps modulated with fs-scale sub-pulses, might be important in various applications, for example in optical coherence tomography^24^ or the efficient generation of the second harmonics^7^.

To generate on demand such double scale temporal structures, we introduce the following objective function that provides a balance between the duration of the envelope of the ACF and the contrast of the coherence peak in the form:

$${{\rm{\psi }}}_{2}=\frac{1}{ACF\,envelope}+Coherence\,spike\,contrast$$

**Figure 5:**
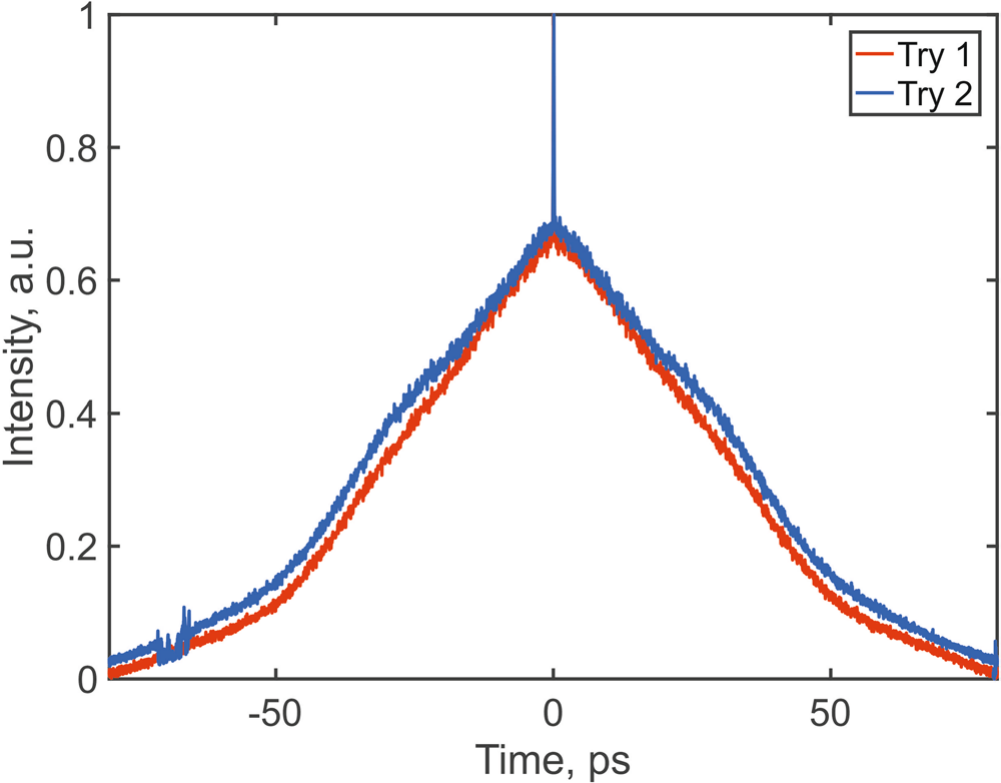
ACF of double scale pulses found by genetic algorithm.

Using this algorithm, the system produced a double scale pulse with an envelope duration of 70 ps and a coherence spike contrast of 0.35 (Fig. 5). Evidently, the degree of coherence of the double scale pulses can be controlled by varying weight of the coherence spike contrast in the objective function.

## Generation of the Pulses With a Maximum Energy

Finally, we have constructed an objective function (${{\rm{\psi }}}_{3}$) to find the pulsed regime with the maximum energy.

$${{\rm{\psi }}}_{3}=RF\,contrast+Power$$

The maximum value of this objective fucntion corresponded to a pulsed regime with an average power of 360 mW, corresponding to a pulse energy of 22.9 nJ.

**Figure 6:**
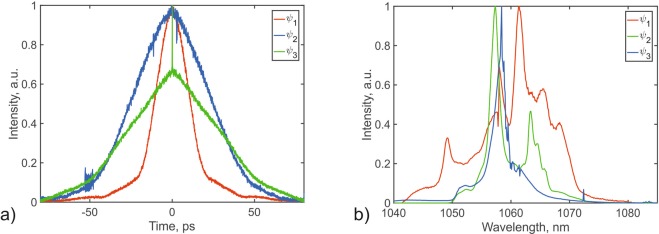
(**a**) ACF and (**b**) Optical Pulse Spectrum found by the genetic algorithm for the three objective functions.

Figure 6 demonstrates that three pulsed regimes, which are qualitatively different from each other, can be generated in a single configuration of a mode-locked fiber laser cavity.

## Conclusions

In conclusion, the present study is the first to demonstrate experimentally the application of machine learning algorithms for the control of the pulsed regimes in the all-normal dispersion figure-eight fibre laser with two independent amplifying fibre loops. This type of laser, with double electronically managed pumps, is especially attractive for implementation of machine learning algorithms. On one hand, there is a large number of possible lasing regimes in the same cavity; on the other hand, these operational regimes are easily controllable electronically by varying the currents of the two pumping diodes. The self-tuning adjustment of the two independent gain levels in the laser cavity and application of various objective functions enabled on-demand generation of: (i) the pulses with the shortest duration, (ii) the highest pulse energy, and (iii) the maximum contrast of coherence that are all possible for the same laser cavity. We have experimentally studied how different objective functions can be used to select the pulse duration, energy and degree of temporal coherence of radiation. Machine learning approaches create possibilities for the practical use of lasing regimes with more complex structures of temporal waveform and spectrum, which would probably not be considered in conventional laser systems due to their limited control. This expands the range of achievable pulse parameters in a given laser cavity and paves the way for controllable generation of much more complex regimes than the traditional mode-locked pulses or CW radiation (see, e.g., discussion in^15–17^). Although the main focus of our experiments was to demonstrate the principle of control and generation of a range of on demand pulses for a particular all-normal dispersion, 8-figure fibre laser with two independent amplifying fibre loops, similar techniques can be applied to a range of laser configurations. We anticipate that ML can provide a new high level of understanding of the lasing regimes that are feasible for a given system configuration at the design and testing stages. ML-based lasers have potential to become self-tuned and extremely robust against various environmental perturbations and hazards. However, this requires further studies and work on simplified methods analysis, as well as real-time processing of the generated signals and subsequent ML-driven feedback to the control elements. We believe that our results show the great potential for control of lasing regimes in fibre lasers and open the way for new designs of pulsed fibre lasers with robust electronics-managed control.

## Methods

### Laser

Our figure-eight mode-locked fibre laser cavity consists of two fibre loops – unidirectional (main) and bidirectional (NALM) ones – connected to each other by a 40/60 coupler. The main loop includes a 70% output coupler and a high-power Faraday isolator that provides unidirectional propagation. Both loops comprise 2.5-m long amplifying sections of double-clad Yb-doped fibres with absorption of 3.9 dB/m at 978 nm. Active fibres are pumped through fibre beam combiners by independently controlled multimode laser diodes with an optical power of up to 4W at a wavelength of 978 nm. All fibres inside the cavity, both passive and active, are polarization-maintaining.

### Measurement system

consists of the APE pulseCheck autocorrelator with scanning range from 120 fs up to 150 ps, the Tektronix RSA 3308B radio-frequency spectrum analyser with 2-Hz resolution for inter-mode beat signal measuring, and the Yokogawa AQ 6375 optical spectrum analyser (OSA) with resolution 0.02 nm.

The signal-to-noise ratio of radio frequency inter-mode beats (RF contrast in RF maps) is measured as a contrast between the background level and the spike at the fundamental frequency. The average optical power is evaluated as the integral of optical spectrum power density measured by OSA and these values were calibrated at several points with an external power meter.

For filtering the ACF and generating the ACF envelop, a low-pass 3-order Butterworth filter with 0.01 (X π rad/sample) cut-off frequency was used. The full-width half maximum of the filtered ACF is used as a measure of pulse duration. The contrast of coherence spike was calculated as a ratio between maximums of unfiltered and filtered ACF.

### Genetic algorithm

To configure the algorithm, the number of individuals in the population, the fraction of elite individuals and the fraction of mutated individuals are used; these parameters are selected empirically. We used following numbers: number individuals in population N_pop_ = 30, Elite fraction F_elit_ = 0.2, Mutation fraction F_mut_ = 0.2. These values provide convergence of algorithm over 10–15 iterations and also avoid clamping on local extremums. In takes 3 second in average to measure 1 pulse regime. The longest part is resetting a power in pumping laser diodes to avoid hysteresis effect of a pulse generation.
